# Spatial Scale Selection for Urban Systems: A Complexity–Heterogeneity Balancing Method

**DOI:** 10.3390/e27111114

**Published:** 2025-10-29

**Authors:** Xiang-Yu Jia, Yitao Yang, Ying-Yue Lv, Erjian Liu, Xiao-Yong Yan

**Affiliations:** 1 Hebei Key Laboratory of Future Urban Intelligent Traffic Management, Beijing Jiaotong University, Beijing 100044, China; xiangyujia@bjtu.edu.cn (X.-Y.J.); 22110237@bjtu.edu.cn (Y.-Y.L.); 2 School of Systems Science, Beijing Jiaotong University, Beijing 100044, China; 3 School of Geography, University of Leeds, Leeds LS2 9JT, UK; y.yang@leeds.ac.uk; 4 Beijing Transport Institute, Beijing 100073, China; erjian@ifisc.uib-csic.es; 5 School of Traffic and Transportation, Shijiazhuang Tiedao University, Shijiazhuang 050043, China

**Keywords:** urban complexity, spatial scale selection, renormalization group, multiscale structural complexity, normalized entropy

## Abstract

Cities are complex systems with socioeconomic activities exhibiting diverse spatial distributions, where selecting an appropriate observation scale is vital for understanding urban complexity. However, the traditional methods for this task are often limited, either because they rely on subjective judgments or lack generalizability before being applied across the diverse functions of a city. To address this issue, we introduce a complexity–heterogeneity balancing method, which employs renormalization group techniques to generate distribution matrices across different scales, striking a balance between complexity and heterogeneity to objectively identify appropriate observation scales. We implement this method on freight, enterprise and restaurant distribution data derived from major Chinese cities to identify their appropriate spatial scales. The results properly reflect the characteristic spatial organization structure of each urban function, meaning that the method provides a robust framework for determining appropriate scales in urban spatial analysis tasks. Our study has potential applications in enhancing the logistics optimization, industrial zoning and commercial planning processes and identifying urban functions and morphological features, thereby contributing to sustainable urban development.

## 1. Introduction

Cities are typical examples of complex systems; they serve as hubs where human socioeconomic activities and resources are densely concentrated [1,2]. They are composed of numerous subsystems that primarily fulfill three functions: supply, demand and transportation [3]. The supply function provides various resources and services for the city, the demand function reflects the dependence of residents and operators on these resources and services [4], and the transportation function ensures the movement of people and materials within the city [5]. Spatial distribution maps are utilized to better comprehend how these subsystems are arranged across a city [6]. However, different results are obtained when observing the spatial distribution maps of various urban subsystems at different scales [7,8,9,10,11]. For example, coarse observation scales are effective for identifying overall layouts and large-scale zoning patterns but overlook the local details and variations within smaller areas [12]. Conversely, fine observation scales excel at revealing localized and individual characteristics but may become overly complex owing to their excessive focus on microstructures, neglecting overall features [13]. Therefore, choosing an appropriate spatial scale that maintains the overall structures and essential characteristics of urban maps while avoiding unnecessary complexity is crucial.

The grid sizes of urban maps typically represent their spatial scales in urban studies. Researchers often divide their study areas into identical square or hexagonal grids, with the selected grid edge length depending on the specific research objectives, the availability of data, and the required precision of the analysis [14]. Common scales include 200 m, 250 m, 500 m, 1000 m, etc. [15,16,17,18,19]. However, these scales are often set subjectively by researchers and lack robust theoretical foundations [20,21,22]. Geographers have proposed methods to address this, for instance, by analyzing trend variations in spatial autocorrelation [23], evaluating estimation errors across scales [24], or balancing noise reduction with the preservation of spatial structure [25]. These studies have made significant progress in terms of determining urban spatial scales, developing various methods and providing theoretical support. However, they all target specific geographic feature element information, thus having limited applicability. Other powerful techniques, such as multifractal analysis [26,27] or wavelet analysis [28], are well-established for characterizing the scaling properties and heterogeneous structures of urban systems across a continuum of scales. However, they are not designed to prescribe an appropriate observation scale for practical application. Consequently, with respect to spatial scale selection, generalizable methods for identifying the appropriate observation scales for various complex systems in cities are lacking.

Recent advancements in complexity science offer new insights for addressing this challenge. Bagrov et al. proposed a method based on renormalization group theory for calculating the multiscale structural complexity (MSC) of matrices [29]. This method generates matrices at different scales through progressive matrix coarse-graining operations. Subsequently, the local complexity at each scale can be obtained by calculating the overlap between adjacent scale matrices. The MSC method can also be applied to characterize the complexity of urban distribution maps [30]. These maps can be represented as pixel matrices, where each pixel cell corresponds to element values (e.g., population density, building height or freight flow values) at a specific spatial location. The spatial scale is determined based on the chosen pixel size. By applying the MSC method, we can assess the complexity of urban maps across various scales. However, MSC cannot fully solve the spatial scale selection problem, as it is necessary to consider both the complexity and the heterogeneity of the examined distribution map. When the initial scale is small, urban maps exhibit pronounced complexity and heterogeneity; as the scale increases, complexity diminishes, whereas homogeneity increases. Multiple pixel cells are mixed into a single item, causing the differences between pixel cells to become less noticeable and increasing the homogeneity of urban maps. Eventually, when the scale increases to the point where only one-pixel block is present, the urban map becomes completely homogeneous. Therefore, determining the ideal observation scale, which should prevent excessive complexity while preserving sufficient heterogeneity, is an important issue. Normalized entropy (NE) is an effective method for measuring heterogeneity [31], and it has been widely applied in various natural and social complex systems. Consequently, the use of both MSC and NE allows us to capture both complexity and heterogeneity information while finding a balance between them.

In this paper, we introduce a complexity–heterogeneity balancing method (CHBM), which simultaneously considers MSC and NE to analyze typical cases of supply, demand and transportation functions in urban systems, demonstrating its applicability across these key urban functions. Our findings reveal that this approach offers a new perspective for selecting appropriate spatial scales, thereby providing valuable tools for urban spatial distribution mapping, transportation zoning and urban morphological feature extraction tasks.

## 2. Results

The cases examined in this study include urban enterprises, restaurants and a freight system, which all play key roles in the supply, demand and transportation functions of cities. First, enterprises function as both producers and consumers in urban systems. As producers, they generate goods and services, create employment opportunities and supply diverse products to satisfy market demands. As consumers, they maintain ongoing requirements for office space, human resources and services provided by other enterprises. We can reveal the geographical distribution characteristics of enterprises in different urban areas by analyzing their quantity distribution data. Second, the restaurant sector reflects urban consumption patterns and the lifestyles of residents. On the supply side, restaurants provide food and beverages to meet the daily needs of residents; on the demand side, they require the continuous procurement of various raw materials, such as vegetables, meat, grains and aquatic products. Analyzing restaurant distribution patterns provides valuable insights into consumption hotspots. Third, the freight system is a crucial link that connects supply and demand, ensuring the circulation and distribution of materials. In cities, freight guarantees the timely supply of production materials and finished products and supports the operational needs of service industries such as food services. Analyzing freight trip distribution data helps us assess the efficiency of logistics and the extent of service coverage.

**Figure 1:**
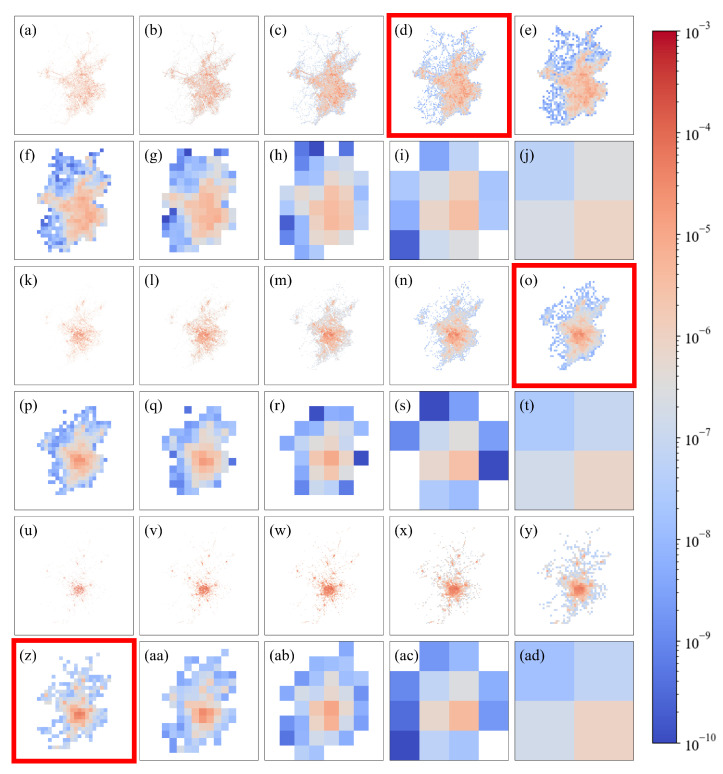
Distribution maps of the freight trips, enterprise quantities and restaurant quantities observed for Beijing at different scales (0.125–64 km). Figures (**a**–**j**) represent the freight trip distributions, Figures (**k**–**t**) represent the enterprise quantity distributions, and Figures (**u**–**ad**) represent the restaurant quantity distributions. The colors reflect the density distribution characteristics of the quantities; i.e., warmer colors indicate higher quantity densities in the corresponding cells, and cooler colors indicate lower quantity densities. The scales correspond to the edge length of each cell, with 0.125 km for subfigures (**a**,**k**,**u**), 0.25 km for (**b**,**l**,**v**), 0.5 km for (**c**,**m**,**w**), 1 km for (**d**,**n**,**x**), 2 km for (**e**,**o**,**y**), 4 km for (**f**,**p**,**z**), 8 km for (**g**,**q**,**aa**), 16 km for (**h**,**r**,**ab**), 32 km for (**i**,**s**,**ac**), and 64 km for (**j**,**t**,**ad**). The subfigures corresponding to the appropriate observation scale are marked with red frames.

We use the CHBM to study the appropriate observation scales for three datasets that include freight trips, enterprise quantities and restaurant distributions for Chinese prefecture-level cities. The freight trip data are sourced from Yang et al. [32], who identified the intercity freight travel chains of heavy trucks using GPS data. The enterprise and restaurant distribution data are sourced from Amap (AutoNavi Software Co., Ltd., Beijing, China; https://www.amap.com; accessed on 15 August 2023), which is a major digital map and navigation service provider in China. We select Beijing as a representative case study to demonstrate the methodology and results. We use a 2048 × 2048 two-dimensional matrix to represent the freight, enterprise and restaurant data of Beijing. Each cell in the matrix represents a square area with a side length of 0.125 km. We visualize these data through color mapping, wherein the pixel values at specific spatial locations correspond to element values, as shown in Figure 1a for the freight case. Furthermore, we apply renormalization group processing to the original matrix according to the following steps.

1. Divide the matrix into larger, nonoverlapping blocks (2 × 2), doubling the spatial scale with each iteration.
2. For each new block, calculate the average of all the pixel values within it to represent that block, thus creating a lower-resolution matrix.
3. To maintain the same number of elements as that contained in the original matrix, re-enlarge the generated matrix to its original size, as shown in Figure 1b.
4. Repeat this process until the matrix is reduced to a single pixel block.

This procedure results in a series of matrices with different scale characteristics, as shown in Figure 1c–j. The approach for processing enterprise and restaurant distribution data follows a similar procedure, as shown in Figure 1k–ad. These subfigures show that when the scale is too small, local details are maximized, but the overall characteristics of the distribution are not obvious because too many details are present. When the scale is too large, the overall characteristics also cannot be reflected due to a lack of heterogeneity. Therefore, we must consider both heterogeneity and complexity to find the appropriate scale.

**Figure 2:**
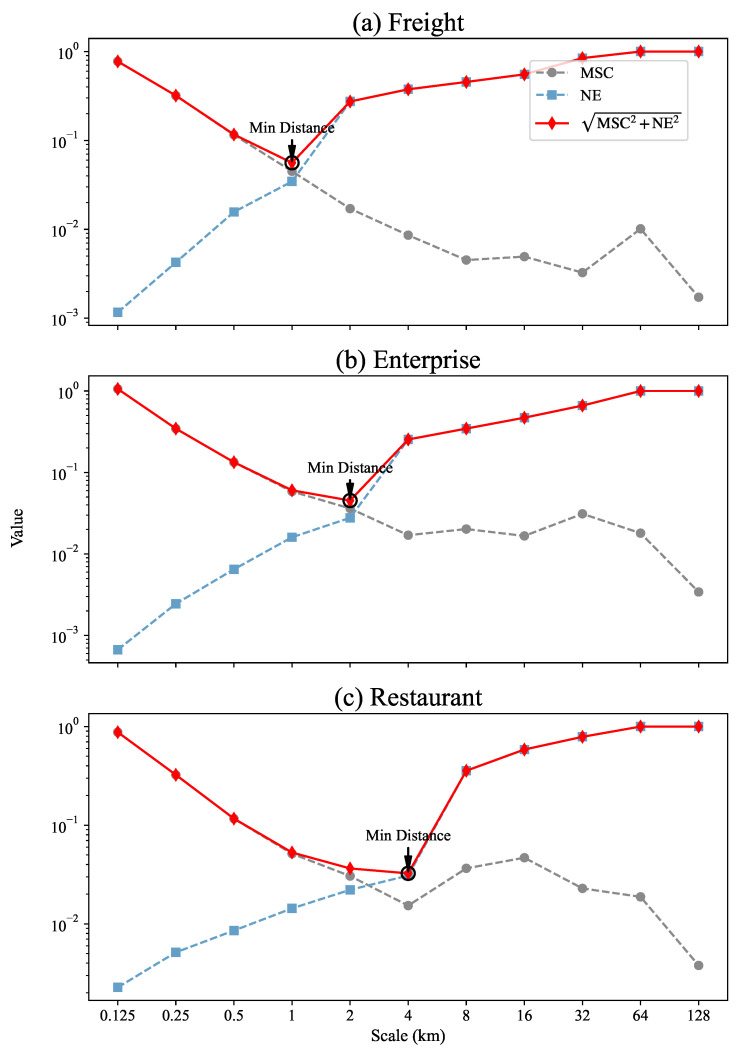
Trends of the MSC values and the NE values with scale changes for the freight trip, enterprise quantity and restaurant quantity distribution maps of Beijing. The dashed circles represent the MSC values, the dashed squares represent the NE values, and the solid diamonds represent the Euclidean distance to the origin in the (MSC, NE) space. The minimum value point of the distance is circled in black.

In terms of complexity, we calculate the MSC for each renormalization process (see Section 4 for details), and these values are shown as circles in Figure 2a. In terms of heterogeneity, we calculate the NE values at different scales, which are shown as squares in Figure 2a. To identify the appropriate observation scale that balances complexity and heterogeneity, we conceptualize their relationship within a two-dimensional feature space where the axes represent MSC and NE. The appropriate scale is then determined by minimizing the Euclidean distance from the origin, which represents an idealized state of zero complexity and entropy (see Section 4 for calculation details). This tradeoff point, marked by a diamond-shaped point in Figure 2, serves as the basis for determining the appropriate observation scale.

For the freight data, at a scale of 1 km (Figure 1d), the Euclidean distance to the origin in the (MSC, NE) space reaches its minimum, as shown in Figure 2a, reflecting a balance between complexity and heterogeneity. At scales smaller than 1 km, the complexity becomes excessively high, whereas at scales larger than 1 km, the distribution tends to be overly homogeneous. The 1 km scale effectively avoids both an excessive focus on fine details, which increases the complexity level, and overly coarse perspectives, which lead to excessive homogeneity. Therefore, we identify 1 km as the appropriate observation scale for analyzing the freight distribution map of Beijing.

Similarly, according to Figure 2b,c, the appropriate observation scales for the enterprise and restaurant distributions of Beijing are 2 km (Figure 1o) and 4 km (Figure 1z), respectively.

**Figure 3:**
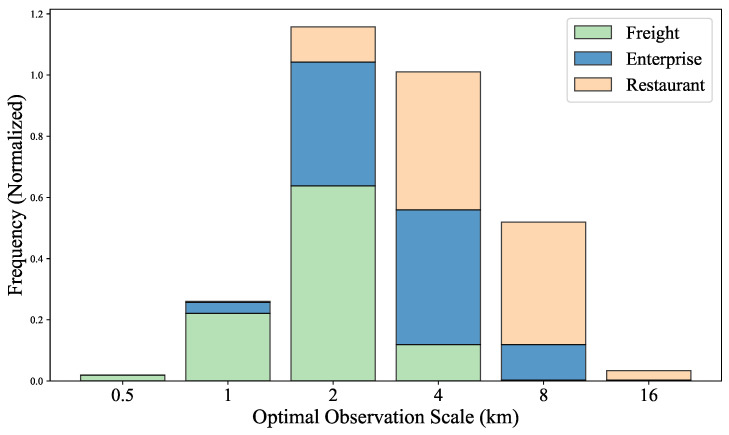
Statistical results regarding the appropriate observation scales determined for freight, enterprise and restaurant distribution data from Chinese prefecture-level cities. The horizontal axis represents the appropriate observation scale (in kilometers), and the vertical axis represents the frequency.

We further calculate the appropriate observation scales for the freight trip, enterprise quantity and restaurant quantity distributions in Chinese prefecture-level cities, with the results shown in Figure 3. The appropriate observation scales differ among the three data types: the freight data are concentrated around 1–2 km, the enterprise data range between 2 and 4 km, and the restaurant data span more broadly from 2 to 8 km.

Freight activities tend to form concentrated clusters in specific areas, such as logistics parks, transportation hubs (e.g., ports, train stations and airports) and major freight corridors [33]. For most cities, scales of 1–2 km (an area of 1–4 km^2^) provide an appropriate observation perspective, enabling the effective identification of cluster effects. This finding is corroborated by empirical data on the actual size of logistics parks. For instance, the seventh National Logistics Park Survey in China found that the vast majority (92.6%) of such parks occupy an area between 0.1 and 3 km^2^ [34], a size range that falls within our observation grid. Furthermore, this scale aligns closely with the literature on transportation zone division scales, where many cities adopt division scales ranging from 0.5 to 2 km^2^ [35,36]. However, the conventional traffic zone division methods largely rely on subjective judgments, i.e., determining the grid edge lengths on the basis of specific transportation planning needs and data availability [37]. In contrast, our proposed method provides an objective framework for selecting appropriate observation scales.

Enterprise activities also form clusters in specific areas, such as business districts, economic development zones and high-tech industrial parks [38]. Previous research has highlighted the spatial heterogeneity of enterprise distributions and their strong association with scale effects [39]. However, these studies focused mainly on macrolevel analyses at the regional or national level and lacked in-depth discussions of the scales of the enterprise distributions within cities. We find that the appropriate observation scales for the enterprise distributions in most cities range between 2 and 4 km, at which point the cluster effects of enterprises can be effectively identified. This study fills the gap in the existing research and provides a scientific basis for the fine-grained management of the enterprise distributions within cities.

For the restaurant industry, the most frequently observed appropriate spatial scales span more broadly from 2 km to 8 km. This reflects the unique spatial clustering logic of the restaurant industry. Restaurants are strongly tied to dense population centers and transportation networks, and these spatial scales closely correspond to the service radius of a typical restaurant [40,41]. The 2 km scale roughly corresponds to a 15–20 min walking distance, which is suitable for reflecting the daily dining travel ranges of consumers [42]; the 4 km scale is closer to a 10 min drive, which is suitable for covering larger restaurant service areas [43]; and scales up to 8 km better capture the long-distance spatial interactions in tourism-dependent cities, where visitors often travel across districts to reach popular dining areas [44]. These appropriate observation scales reflect the spatial differences exhibited by the development trend of the urban restaurant industry, providing a new perspective for understanding the lifestyles and economic development processes of different cities.

In conclusion, our study reveals that different urban functions require distinct observation scales, as demonstrated through our analysis of freight trip, enterprise quantity and restaurant quantity distributions. Through an analysis of empirical data, we establish the fact that the CHBM offers an innovative framework for analyzing urban spatial structures across various functional domains, advancing our understanding of the multiscale nature of urban systems.

## 3. Discussion

Cities, as complex systems with concentrated socioeconomic activities, require carefully selected scales for mapping spatial distributions, which shapes our understanding of urban complexity [10,45]. Traditionally, subjective judgment has dominated the scale selection methods for urban maps [37], lacking a unified theoretical basis and struggling to meet diverse functional needs. The CHBM addresses this issue by using renormalization group techniques to generate distribution matrices at varying scales, balancing complexity and heterogeneity to objectively determine appropriate observation scales. For Beijing, the appropriate observation scales are 1 km for the freight trip distribution, 2 km for the enterprise quantity distribution, and 4 km for the restaurant quantity distribution. These scales effectively capture the spatial patterns of the logistics hubs, polycentric business districts and dining distribution of the city, reflecting their distinct urban functional structures. These insights provide a reliable reference for urban planning in Beijing, facilitating the optimization of logistics networks, industrial zoning schemes and commercial spatial arrangements. An analysis of Chinese prefecture-level cities reveals appropriate scales of 1–2 km for freight, 2–4 km for enterprises and 2–8 km for restaurants based on their clustering patterns and accessibility needs. Variations across cities highlight their unique development and socioeconomic contexts, enabling quantitative city classification work and comparative studies.

The significance of the CHBM lies not only in its suitability as a reference tool for scale selection but also in its potential applicability to urban feature extraction tasks, identifying different urban functions in a fine-grained manner. The CHBM balance point identifies the scale at which a system’s structure (e.g., freight hubs, enterprise clusters) becomes maximally coherent and discernible from both micro-level stochastic noise (at finer scales) and macro-level over-simplification (at coarser scales). This provides a reliable basis for urban planning, shifting policy from imposing arbitrary, top-down administrative grids to aligning interventions with the endogenous functional scales of the city’s own socioeconomic processes. Additionally, our method can effectively analyze urban spatial structures and morphological characteristics, including urban compactness [46], polycentric layouts [47] and sprawl tendencies [48]. By extracting urban features at different scales, the spatial patterns of urban development can be deeply quantified, more keenly capturing the subtle pattern changes exhibited by urban development and providing valuable support for the scientific delineation of urban renewal and growth boundaries.

Despite the significant progress made in this research, several limitations remain. The current analysis focuses on three functions, which include the freight system, enterprises and restaurants, and the broader applicability of the proposed method needs to be tested through validations involving more urban elements (such as residential areas and green spaces). Another limitation is our static analysis, which does not account for the fact that the appropriate spatial scale is time-variant. Future research could address this spatio-temporal coupling by extending the renormalization group procedure to three dimensions to find a simultaneous optimum. Additionally, the research relies on a square grid division. This is because the current CHBM relies on iterative 2 × 2 block coarse-graining calculations; future studies could explore the development of a generalized renormalization procedure for hexagonal or other grid types. Moreover, the research subjects are limited to Chinese prefecture-level cities without covering a global range, limiting the exploration of the scale differences between cities with different socioeconomic and cultural backgrounds. For example, European and American cities may present unique spatial characteristics due to their different urbanization histories and planning concepts [49]. Through these improvements, the CHBM is expected to play a greater role in research on urban complexity and heterogeneity, providing broader support for sustainable urban development strategies worldwide. By enabling urban spatial patterns to be more precisely characterized, this method can help cities optimize their resource allocation schemes, reduce their ecological footprints, and enhance the efficiency of their urban systems, ultimately contributing to more sustainable urban futures.

## 4. Methods

### 4.1. MSC Calculation Method

In accordance with Bagrov’s approach [29], the MSC of a pattern can be defined within the normalization group flow framework. In this framework, a pattern can be regarded as a function f(x) defined over a certain domain *D*. For example, in grayscale images or spatial data, this function manifests as a two-dimensional matrix. The MSC calculation process can thus be divided into the following key steps.

1. Matrix Initialization. The starting matrix with a size of L×L is divided into many blocks with sizes of Λ×Λ, where Λ=2 in this study.
2. Iterative Coarse-Graining. In each iteration, the next-scale matrix is generated by calculating the average value of the elements within each block. This process is formulated as follows:
  (1) $$s_{ij}^{\left(k\right)}=\frac{1}{\Lambda^{2}}\sum_{l}\sum_{m}s_{\Lambda i+m,\Lambda j+l}^{\left(k-1\right)},$$
  where *l* and *m* are matrix indices belonging to the same block, and *k* denotes the number of iterations. This process is repeated multiple times, resulting in renormalized matrices at different resolutions.
3. Overlap Calculation. To compute the overlap between the matrices separated by one renormalization group step, each generated matrix is first upscaled back to its original size so that the number of elements remains consistent. The formula for computing the degree of overlap is as follows:
  (2) $$O_{k,k-1}=\frac{1}{L_{k-1}^{2}}\sum_{i=1}^{L_{k}}\sum_{j=1}^{L_{k}}s_{ij}^{\left(k\right)}\cdot \sum_{m=1}^{\Lambda }\sum_{l=1}^{\Lambda }s_{\Lambda i+m,\Lambda j+l}^{\left(k-1\right)}.$$
  Simplifying this formula yields the following:
  (3) $$O_{k,k-1}=\frac{\Lambda^{2}}{L_{k-1}^{2}}\sum_{i=1}^{L_{k}}\sum_{j=1}^{L_{k}}{\left(s_{ij}^{\left(k\right)}\right)}^{2}=O_{k,k}.$$
4. Structural Complexity Computation. At each scale *k*, the structural complexity $C_{k}$ (i.e., the MSC at scale *k*) is defined as the scaled difference between overlaps:
  (4) $$C_{k}=\alpha \cdot \left|O_{k+1,k}-\frac{1}{2}\left(O_{k,k}+O_{k+1,k+1}\right)\right|.$$
  To ensure a balanced comparison between these two distinct metrics, we introduce a scaling factor α, which is defined as the product of the initial matrix dimensionality and the number of renormalization steps. This normalization step is crucial, as it scales the MSC to a comparable order of magnitude relative to that of the NE. Without it, the metric with the inherently larger numerical value would dominate the distance calculation, preventing a true balance between complexity and heterogeneity from being achieved. This ensures that both metrics contribute meaningfully to the identification of the tradeoff point.

This measure allows us to assess how the structures of urban patterns evolve across scales, identifying the scale at which the examined system transitions from being highly complex to overly homogeneous.

### 4.2. NE Calculation Method

NE, which is derived from Shannon entropy in information theory [31], provides an effective way to quantify the uniformity or diversity of spatial distributions. It is widely used in analyses of complex systems. Its computation process involves the following steps.

1. Probability Distribution. The matrix $s^{\left(k\right)}$ at each scale is flattened into a one-dimensional array, and the probability distribution of its elements is calculated. Assuming that the matrix contains *N* elements, the probability of the *i*-th element is defined as
  (5) $$p_{i}^{\left(k\right)}=\frac{s_{i}^{\left(k\right)}}{\sum_{j=1}^{N}s_{j}^{\left(k\right)}},$$
  where $s_{i}^{\left(k\right)}$ is the value of the *i*-th element in the matrix and $\sum_{j=1}^{N}s_{j}^{\left(k\right)}$ is the total sum of all the elements.
2. Entropy Calculation. On the basis of the probability distribution $p_{i}^{\left(k\right)}$, the Shannon entropy $H^{\left(k\right)}$ is computed:
  (6) $$H^{\left(k\right)}=-\sum_{i=1}^{N}p_{i}^{\left(k\right)}\log_{2}p_{i}^{\left(k\right)}.$$
  The Shannon entropy $H^{\left(k\right)}$ reflects the degree of heterogeneity exhibited by the distribution. When all $p_{i}^{\left(k\right)}$ values are equal (i.e., the distribution is completely uniform), $H^{\left(k\right)}$ reaches its maximum. When one $p_{i}^{\left(k\right)}=1$ and all others are zero (i.e., the distribution is completely concentrated), $H^{\left(k\right)}=0$.
3. Normalization. To ensure comparability across different scales and datasets, the Shannon entropy is normalized. The NE is defined as shown below:
  (7) $$H_{n}^{\left(k\right)}=\frac{H^{\left(k\right)}}{\log_{2}N}.$$
  Here, $\log_{2}N$ is the maximum possible entropy corresponding to a completely uniform distribution where all $p_{i}^{\left(k\right)}$ values are equal. The value of $H_{n}^{\left(k\right)}$ ranges from 0 to 1, where 0 indicates maximum heterogeneity and 1 indicates a completely homogeneous distribution.

By combining both the MSC and the NE, the CHBM achieves a balanced assessment of the complexity and heterogeneity exhibited across different spatial scales. The appropriate observation scale is determined by minimizing the distance $\sqrt{{\left(C_{k}\right)}^{2}+{\left(H_{n}^{\left(k\right)}\right)}^{2}}$ to the origin of the feature space, which represents an ideal state of zero complexity and entropy. This formulation represents the standard Euclidean distance, which implicitly assigns equal weight to both complexity and heterogeneity, and serves as our objective function. Furthermore, we conduct a sensitivity analysis to test the robustness of the appropriate scale selection. This analysis involves systematically varying the weights in the objective function, defined as $\sqrt{w\cdot {\left(C_{k}\right)}^{2}+\left(1-w\right)\cdot {\left(H_{n}^{\left(k\right)}\right)}^{2}}$. Specifically, the weight *w* is varied from 0.1 to 0.9 in increments of 0.1. We define robustness as the frequency of the most frequently occurring appropriate scale within this tested range of weights for a given city and data type. Across all cities and data types analyzed, the CHBM yields an average robustness of 79.4%. This indicates that the identified appropriate scale is largely insensitive to the specific weighting of the objective function’s components, confirming the stability and reliability of our approach.

## Data Availability

The data that support the findings of this study are available from the corresponding author upon reasonable request.
